# Genetic control of malaria transmission: a salivary gland-centric perspective^☆^

**DOI:** 10.1016/j.cois.2025.101462

**Published:** 2025-11-17

**Authors:** Sabina Eze, Valerie T Nguyen, Alec Morvay, Yoosook Lee, Bianca Correa Burini

**Affiliations:** Florida Medical Entomology Laboratory, IFAS, University of Florida, Vero Beach, FL 32962, USA

## Abstract

Mosquitoes are the deadliest organisms on Earth, as they transmit a wide range of medically important diseases, posing a major public health threat worldwide. Controlling vector-borne diseases presents numerous challenges, and the alarming rise in their incidence underscores the urgent need for innovative strategies, with genetic control offering a promising approach. Genetic engineering of mosquitoes requires a profound understanding of mosquito physiology and molecular aspects of host–pathogen interactions. Over the years, several stages of the *Plasmodium* parasite development within the mosquito have been extensively studied, with many molecular mechanisms successfully elucidated. However, important features of the parasite journey remain unclear, particularly the sporozoite’s ability to recognize and invade the salivary glands. This process involves a complex interplay of proteins and other molecules, yet much remains to be discovered about the precise mechanisms at play. Advancing our knowledge of this critical step will be instrumental in designing more effective transgenes for genetic control strategies, particularly those aimed at mosquito population replacement.

## Introduction

Mosquito-borne diseases remain a major global health threat, transmitting malaria, dengue, and others. In 2024, malaria alone caused an estimated 263 million cases and 597 000 deaths worldwide [1]. Despite widespread use of traditional control methods, disease burden continues to rise due to insecticide resistance and climate change [2,3], highlighting the need for novel tools to control disease transmission.

Inside the mosquito, *Plasmodium* undergoes multiple developmental transitions to reach the salivary glands and become infectious [4]. Among these, ookinete invasion of the midgut and oocyst formation are the best studied, uncovering molecular mechanisms at a major bottleneck where parasite numbers drop sharply. The reduction of parasites makes the midgut stage an attractive target for genetic interventions aimed at blocking transmission [5].

In contrast, sporozoites have historically been considered less appealing as a target because their numbers increase substantially after oocyst rupture, releasing large quantities of parasites into the mosquito hemolymph and therefore remain comparatively understudied. Only a few promoters and effector molecules have been identified that can specifically target this tissue, even though salivary gland invasion itself presents a substantial bottleneck, since only a small proportion of sporozoites are able to successfully penetrate the gland and make it to the saliva [6,7]. Understanding the molecular landscape of the mosquito salivary glands could yield promoters capable of targeting both noninfectious and infectious parasite stages (i.e. before and after salivary gland invasion). This makes the salivary gland an important, yet underutilized, target for designing new transgenes. Here, we emphasize the importance of further investigating host–pathogen interaction, as well as reviewing the progress made to date in this area.

## Salivary gland promoters

**Figure 1:**
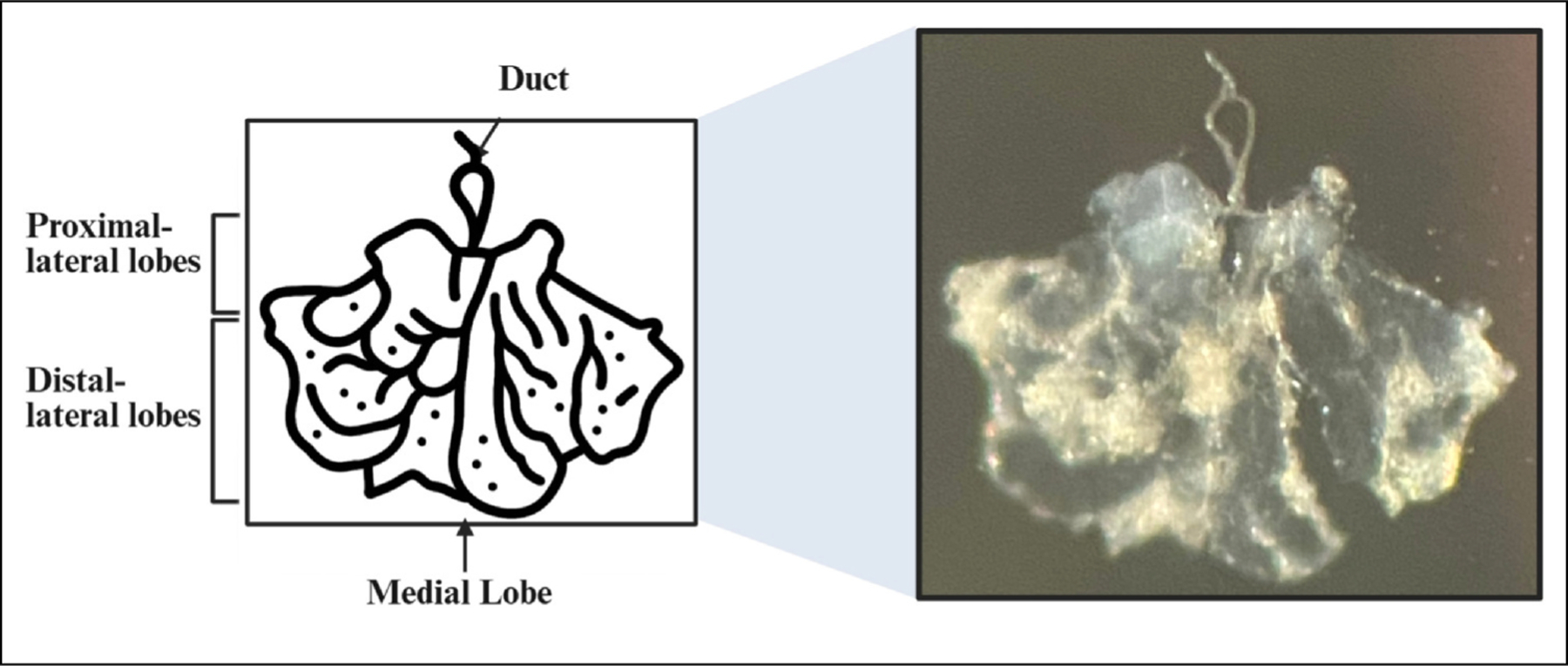
Anatomical labeling of the mosquito salivary gland. The left panel shows a schematic representation of the mosquito salivary gland, illustrating the distal, proximal, and lateral lobes. Distal refers to the portion located away from the salivary gland duct, whereas proximal refers to the portion near the duct. The medial lobe corresponds to the vertical midline of the gland, while the lateral lobes are positioned farthest from this midline. The right panel presents a corresponding image of the dissected gland. The image was acquired using a Leica S9 stereomicroscope at 40× magnification, showing the same anatomical regions.

The mosquito salivary gland is a specialized tissue that secretes a complex cocktail of pharmacologically active components in the saliva, designed to overcome the host’s hemostatic defenses while modulating immune responses and reducing inflammation [8,9]. The mosquito salivary gland is a paired, lobed organ located in the thorax, composed of distinct lobes connected by ducts that channel saliva into the proboscis (Figure 1) [10]. Their surface is lined with a basal lamina, with the two lateral lobes consisting of proximal, intermediate, and distal regions, and the medial lobe consisting of a neck and distal region [11,12].

Mosquito salivary gland regions express distinct proteins in specialized cells, reflecting their roles in feeding and pathogen transmission [11]. The regulation of protein expression depends on promoters and DNA sequences upstream of the 5′ untranslated regions (UTR). Promoters often contain core elements like the TATA box or Initiator [13]. These elements in the promoter region initiate transcription and control when, where, and how much a gene is expressed. The characterization of salivary gland promoters facilitates the expression of molecules that interfere with sporozoite development in transgenic mosquitoes. We emphasize that this is particularly intriguing given that numerous studies have generated transgenic mosquito lines expressing antiparasitic genes targeting midgut or hemolymph stages, markedly reducing the number of sporozoites reaching the salivary glands but failing to entirely prevent their penetration, rendering them suboptimal for genetic control [14,15]. The development of novel salivary gland-specific promoters coupled with new effector genes could complement these existing strategies, eliminating residual parasites that evade earlier barriers and ultimately achieving complete transmission blockage.

Classical promoter characterization in mosquitoes involves cloning the candidate sequence upstream of a reporter gene (e.g. green fluorescent protein [GFP], luciferase) and introducing it into embryos via micro-injection, often using transposable elements for stable genome integration [16]. Promoter activity is then assessed by analyzing the reporter’s spatial and temporal expression across various tissues, developmental stages, or physiological conditions, using fluorescence microscopy and histochemical staining, which reveals its strength and specificity *in vivo*.

So far, seven promoters have been characterized in mosquitoes, most from *Anopheles gambiae* and *Aedes aegypti*.

### Apyrase promoter

Apyrase is a salivary enzyme that prevents clotting by hydrolyzing ADP and ATP, enabling efficient feeding. It also promotes fibrin degradation and supports malaria transmission [17,18]. The apyrase promoter was among the first characterized in mosquitoes. In *Ae. aegypti*, it drove luciferase expression specifically in female salivary glands in the medial and distal lateral lobes, mirroring native gene activity [19]. Similarly, in *An. gambiae*, the apyrase promoter showed salivary gland–restricted expression in some transgenic lines, though variability arose due to position effects and multiple insertions [17,20,21].

### Maltase-I promoter

Maltase-I is a mosquito enzyme that hydrolyzes maltose into glucose, supporting sugar digestion and energy production [22]. Its promoter, characterized alongside apyrase in *Ae. aegypti* [21], drives expression predominantly in the proximal lateral lobes of female salivary glands. While a shorter fragment of 650 base pairs (bp) immediately upstream of the initiation codon directed constitutive expression of the luciferase reporter gene in cultured cells, approximately 1500 bp of the promoter sequence was used to direct developmental-, sex-, and tissue-specific expression of the reporter gene in patterns identical to those of the endogenous *maltase I* gene [21].

### Anopheline antiplatelet protein promoter

Anopheline antiplatelet protein (AAPP) is an antic-oagulant that facilitates mosquito blood feeding by binding collagen at the bite site, preventing platelet adhesion and activation [23,24]. Its promoter, characterized in *Anopheles stephensi* and *An. gambiae*, drives strong, female-specific expression mainly in the distal lateral lobes, with minimal or no expression elsewhere. In transgenic mosquitoes, it has been used to express reporter genes like DsRed, confirming localized activity, and to express anti-AAPP antibody fragments, which reduced feeding success, blood meal size, and fecundity [25–27].

### Triple functional domain protein promoter

Triple functional domain protein (TRIO) in *An. gambiae* is a salivary gland protein secreted into saliva that aids sporozoite movement in the host dermis and contributes to malaria transmission [28]. Its promoter drives expression mainly in the medial lobe apex of female salivary glands, with robust reporter activity in adults. TRIO induces variable expression levels throughout development, including larval, pupal, and adult stages, with occasional expression occurring in Malpighian tubules and antennae [25].

### D7r2 and D7r4

The D7r2 and D7r4 proteins are members of the D7 family and are found abundantly in mosquito saliva. These proteins function primarily as anti-inflammatory and antihemostatic agents by binding host biogenic amines such as serotonin, norepinephrine, and hista-mine. This interaction inhibits vasoconstriction, platelet aggregation, and inflammatory responses at the bite site, thereby facilitating blood feeding and potentially aiding parasite transmission [29]. Promoter characterization of the *D7r2* and *D7r4* was performed in transgenic *An. gambiae*, displaying female-specific, salivary gland–restricted expression of reporter genes [30]. D7r2 and D7r4 promoters drive expression in the distal lateral and medial lobes of female salivary glands.

### 30K Promoter

The 30K are abundant salivary proteins initially identified as allergens involved in mosquito bites. These proteins, including members like 30Ka and 30Kb, play important roles in blood feeding by inhibiting platelet aggregation and adhesion, primarily through binding to collagen at the bite site. This antihemostatic function helps mosquitoes feed efficiently on blood [31,32]. Promoter characterization of the 30K was performed in *Ae. aegypti*, revealing that the 30K promoter is bidirectional, simultaneously driving expression of 30Ka and 30Kb, from closely spaced start sites, driving strong, female-specific, salivary gland expression. In transgenic mosquitoes, reporter gene expression was confined to the salivary glands of adult females in the distal lateral lobes, with transcripts sometimes detected in other tissues [31].

Some attempts were made to characterize the *saglin* promoter, a member of the SG1 protein family and a determinant for sporozoite invasion of salivary glands in *An. gambiae* [25]. The *saglin* lies within an array of four salivary gland-specific genes separated by short inter-genic regions. The cloning of a 200 bp upstream sequence of SG1 did not include the *saglin* promoter, indicating *saglin* promoters are probably shared and positioned outside the cloned fragment. Therefore, the *saglin* promoter is not currently available [25]. Table 1 summarizes characterized salivary gland promoters for directing gene expression in mosquitoes.

## Genetic barriers to sporozoite invasion

Effector molecules are small molecules or proteins that modulate biological systems and interfere with malaria parasites at various stages of development. In engineered mosquitoes, specifically focusing on the sporozoite stage, effector molecules are expressed in the hemolymph or salivary glands in gain-of-function strategies, and are implemented using transposable elements to introduce genes into the mosquito genome [33]. Loss-of-function approaches, such as gene knockouts using CRISPR/Cas9, aim to block parasite invasion in the salivary glands [34]. Effector molecules fall into the category of immune enhancement, immune activators, or host factors interference to block parasite development and have been reviewed elsewhere [35].

### Transgene expression

Recent advances in transgenic mosquito research have demonstrated that sporozoite invasion can be effectively blocked through transgene expression (gain-of-function) approaches. One of the first reported examples of refractory mosquitoes was the expression of the SM1 peptide in transgenic *An. stephensi*, which drastically reduced sporozoite invasion [36]. Expression of a modified *Plasmodium falciparum* circumsporozoite protein (CSP)-targeting single-chain antibody (m2A10) further advanced this concept, greatly reducing the parasite’s ability to reach and invade the salivary glands of *An. stephensi* [14]. Multistage effectors, combining single-chain antibodies with antimicrobial peptides, acted in both the midgut and salivary glands, reducing sporozoite prevalence and oocyst formation, and produced the first transgenic mosquitoes nearly fully refractory to *P. falciparum* [5]. Recently, CRISPR-Cas9 was used to knock in the 2A10 antibody in frame with the nutrient transporter Lipophorin, significantly reducing transmission, consistent with previous reports [5,14,26,37]. Most of these molecules act against sporozoites circulating in the hemolymph before they penetrate the salivary glands. In fact, most effector strategies tested so far have focused on targeting sporozoites during their journey through hemolymph toward salivary glands.

Only a few studies have concentrated on blocking sporozoites after they enter the salivary glands. The 2A10 antibody was expressed in transgenic *An. stephensi* in the secretory cavities and ducts of the salivary glands, and the infectivity of the mosquitoes to mice was strongly impaired [26]. The expression of the human-derived molecule (huPAI-1) in *An. stephensi* interfered with parasite use of host proteolytic systems during gland invasion and reduced infection rates by more than 60% [38]. PAI-1 functions by blocking the activation of plasminogen, thereby preventing the breakdown of extracellular barriers [38]. This disruption slows the parasite’s movement and spread, ultimately reducing salivary gland colonization. These data highlight that progress has been made in developing molecules with different modes of action to target sporozoites. However, there remains ample opportunity to explore new targets within the salivary gland, such as direct killing of sporozoites.

### Mutant mosquitoes

To date, six mosquito factors have been identified as potential receptors or key molecules involved in sporozoite entry into the salivary gland: Salivary Gland Surface Protein 1 (SGS1) [15], Saglin [39], Circumsporozoite Binding Protein (CSPBP) [40], *Plasmodium*-Responsive Salivary 1 (PRS1) [41], Epithelial Serine Protease (ESP) [42], and Glucose Transporter [43]. Application of reverse genetics resulted in a reduction in sporozoite numbers within the salivary glands for all factors. Among them, only SGS1 [44] and Saglin [45] have been fully knocked out to experimentally assess their roles, whereas the remaining factors were investigated using RNAi-mediated knockdown [46]. Notably, recent studies revealed that Saglin knockout does not specifically affect sporozoite invasion, as the observed decrease in sporozoite burden mirrored the reduced oocyst numbers, indicating that this protein is not directly involved in salivary gland entry [25,41]. This finding leaves only five factors as currently validated candidates for mediating sporozoite invasion.

Importantly, removal or disruption of any of these remaining factors did not result in a complete abolition of sporozoite transmission [5,14,15,25,26,37–43], suggesting that multiple redundant mechanisms may regulate salivary gland invasion. Consequently, alternative approaches are required to block sporozoite entry. Nevertheless, these studies are valuable, as understanding host–pathogen interactions is crucial for designing genetic strategies that impede transmission.

## Conclusion

**Figure 2:**
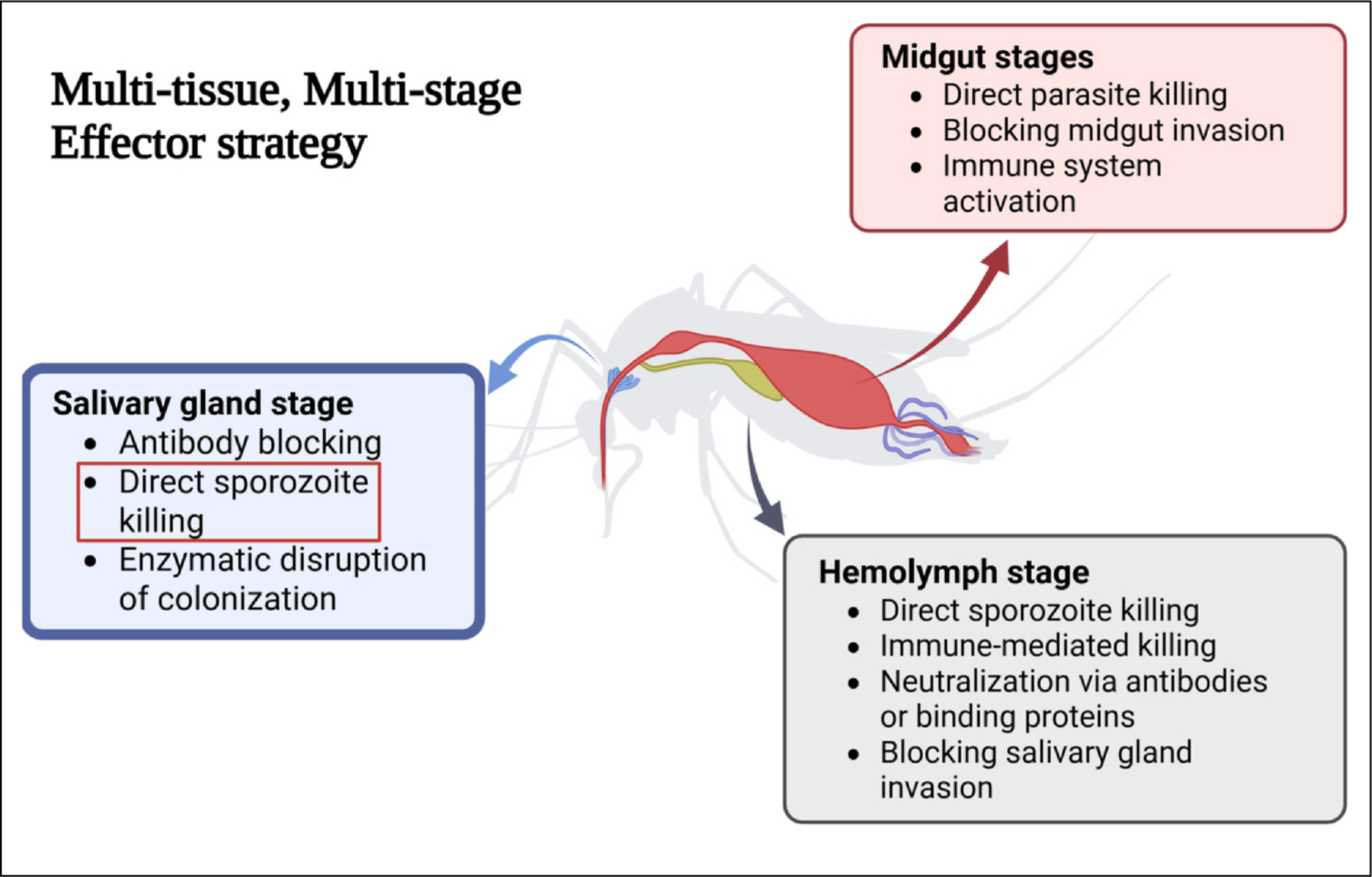
A multi-tissue, multi-stage effector strategy to achieve complete refractoriness to *Plasmodium* in transgenic mosquitoes. Schematic representation of the proposed multi-tissue, multi-stage effector strategy designed to block *Plasmodium* development at successive stages within the mosquito vector. Distinct effector molecules can be expressed in key tissues, including the midgut, hemolymph, and salivary glands, to target parasites as they progress from ookinetes and oocysts to sporozoites. In the midgut, effectors may disrupt parasite invasion or induce the lysis of gametocytes, zygotes, ookinetes, and early oocysts (midgut stages, as shown in the light red box). In the hemolymph, immune activators, antibodies, and antimicrobial peptides can neutralize or eliminate migrating sporozoites (as shown in the gray box). The salivary glands, however, remain the least explored tissue for effector expression, and they are highlighted in blue. Here, we outlined in red a proposed and previously untested approach: direct sporozoite killing within the salivary glands, as a critical step toward achieving full refractoriness and the complete abolition of parasite transmission.

Although salivary gland promoters and effector molecules targeting sporozoites have shown promise, most advances in this area are dated, and renewed efforts are urgently needed. No single transgenic line has yet achieved complete transmission blocking, suggesting that a multistep strategy will be required. Adding effectors that act within the salivary glands to eliminate the residual sporozoites that escape earlier barriers could provide the redundancy needed for full refractoriness. Ultimately, integrating multiple effector genes that target different stages of parasite development will be key to advancing genetic strategies toward malaria elimination (Figure 2).

## Figures and Tables

**Table 1:** Summary of salivary gland promoters: expression sites, protein function, and associated species.

Salivary gland promoters	Expression site	Protein function	Species	References
Apyrase	Preferentially expressed in females. Medial and distal-lateral lobes.	Inhibition of platelet aggregation Regulation of hemostasis	*Aedes aegypti Anopheles gambiae*	[17,19–21]
Maltase-I	Both male and female adult mosquitoes. In males, it is expressed throughout the entire gland, whereas in females, it is expressed in the proximal-lateral lobes.	Critical for sugar digestion and energy production	*Aedes aegypti*	[21]
*Anopheles* Antiplatelet protein AAPP	Distal lateral lobes. Upregulated following a blood meal	Inhibition of platelet aggregation in the blood.	*Anopheles stephensi Anopheles gambiae*	[25–27]
Triple functional domain protein (TRIO)	Medial Lobe	Involvement in parasite transmission	*Anopheles gambiae*	[25]
D7r2 and D7r4	Exclusively expressed in females. Distal lateral lobes and Medial lobes.	Anti-inflammatory and antihemostatic agents. Binds to host biogenic amines such as serotonin, norepinephrine, and histamine	*Anopheles gambiae*	[30]
30K	Distal lateral lobes	Inhibitors of platelet aggregation and adhesion, primarily through binding to collagen at the bite site	*Aedes aegypti*	[31]

## Data Availability

No data were used for the research described in the article.
